# Effects of the supplementation of a calcium soap containing medium‐chain fatty acids on the fecal microbiota of pigs, lactating cows, and calves

**DOI:** 10.1111/asj.13636

**Published:** 2021-10-05

**Authors:** Hiroki Matsui, Taichi Imai, Makoto Kondo, Tomomi Ban‐Tokuda, Yutaka Yamada

**Affiliations:** ^1^ Graduate School of Bioresources Mie University Tsu Japan; ^2^ Agromedic group, R&D Lab. Yuka Sangyo Co., Ltd Amagasaki Japan

**Keywords:** *Campylobacter jejuni*, Japanese Black calf, lactating cow, pig, *Salmonella* spp

## Abstract

Medium‐chain fatty acids (MCFAs) have antialgal, antibacterial, antifungal, antiprotozoan, and antiviral activities. However, antibacterial activities of MCFAs in the hindgut of pigs and cattle are still unknown. We report the effects of the supplementation of MCFAs on fecal bacteria of pigs, lactating cows, and Japanese Black calves. *Lactobacillus* spp., *Bifidobacterium* spp., *eaeA*(+) *Escherichia coli* , *Salmonella* spp., *Campylobacter jejuni* , and *Clostridium perfringens* in the feces of animals were quantified by real‐time PCR assay. There was no significant increase or decrease in *Lactobacillus* spp. and *Bifidobacterium* spp. in the three animals. In the pig feces, *eaeA*(+) *E. coli* was reduced to less than a third in the treatment group (*P* < 0.01). *C. jejuni* in the pig feces was also significantly less in the treatment group compared with the control (*P* < 0.01). In the lactating cow, *eaeA*(+) *E. coli* was reduced to one fifth of that in the control (*P* < 0.01). *Salmonella* spp. was halved in calf feces (*P* < 0.01). Thus, a reduction in Gram‐negative pathogenic bacteria was observed. In conclusion, supplementation of a MCFA calcium soap in the diet would be beneficial to growing pigs, lactating cow, and calves by reducing pathogenic bacteria.

## 1 INTRODUCTION

Medium‐chain fatty acids (MCFAs) have 8 to 12 carbon atoms and are mainly found in coconut and palm kernel oils. MCFAs display unique nutritional characteristics that are different from those of long‐chain fatty acids. Owing to the higher water solubility of MCFAs compared with long‐chain fatty acids, triacylglycerol resynthesis and transport by chylomicrons is not necessary, such that MCFAs can be absorbed directly through the portal vein to the liver (Liu et al., 2011). MCFAs are predominantly subject to metabolism into carbon dioxide, acetate, and ketones through β‐oxidation and can be quickly utilized as energy without being stored as fat (Liu et al., 2011).

The detailed functions of supplying MCFAs in piglet diets can be characterized in a scale‐up experiment. Hanczakowska et al. (2011) reported that (based on 252 newborn piglets) the addition of 0.1% (1 g/kg) octanoic acid (C8:0) or decanoic acid (C10:0) increased the body weight at 84 days by 14% to 22%, increased the average daily weight gain by 15% to 23%, reduced the absolute value of the feed‐to gain ratio by 0.2–0.4, increased the crude protein and the crude fat apparent digestibility by 5%, and increased the crude fiber apparent digestibility by 10% to 13% (Hanczakowska et al., 2011). The addition of octanoic acid (C8:0) or decanoic acid (C10:0) also increased the intestinal villus height and crypt depth, the ratio of villus height/crypt depth, with the decanoic acid (C10:0) showing more pronounced results. A few studies have also reported utilization of MCFAs in the diets of cattle (Piepers & De Vliegher, 2013) and bulls (Panyakaew et al., 2013).

In vitro studies have showed that MCFAs have antialgal, antibacterial, antifungal, antiprotozoan, and antiviral activities (Desbois & Smith, 2010). Their antibacterial mechanisms have not been clarified, but the basic mechanisms are due to the specific amphiphilic chemical structure of MCFAs, which destroys the cell membrane leading to leakage of intracellular materials and bacterial death (Kim & Rhee, 2013). Some antibiotics have commonly been used as growth promoters in animal feed (Barton, 2000). However, in 2006, the use of antibiotics as growth promoters was forbidden in the EU (Chen et al., 2005), and an expansion of this policy to other countries can now be expected. Intensive research is focused on the development of alternative strategies with the aim of maintaining animal health and performance (Bomba et al., 2006; Castillo et al., 2008; Chen et al., 2005). MCFAs are proposed as an alternative to in‐feed antibiotics, to be used for growth promotion, and even for the prevention and treatment of gastrointestinal diseases (Decuypere & Dierick, 2003). However, antibacterial activities of MCFAs in the hindgut of pigs and cattle are still unknown. Furthermore, effects of MCFAs on health‐promoting bacteria in the hindgut of pigs and cattle are not known.

In the present study, the effects of supplementation of a MCFA calcium soap in diets of pigs and cattle on pathogenic and health‐promoting bacteria in the feces of pigs and cattle were measured.

## 2 MATERIALS AND METHODS

### 2.1 Animals and diets

Twelve crossbreed, growing, castrated pigs (Landrace × Large White × Duroc) (average body weight: 43.6 kg) were used in this study. They were evenly divided into control and treatment. Body weight of animals in control and treatment was almost same. In the control, pigs were fed a commercial growing diet without antibiotics. The diet consisted of the following ingredients on dry matter basis: 55.2% maize, 20% milo, 16.0% defatted rice bran, 6.0% fish meal, 0.95% calcium carbonate, 0.95% dicalcium phosphate, 0.30% salt, 0. 20% vitamin B complex, 0. 20% vitamin ADE, and 0.20% trace minerals. GE of the diet was 4.06 Mcal/kg. In the treatment group, pigs were fed the commercial growing diet in a 94:6 ratio with MCFA calcium soap (Starmate; Yuka Sangyo Co., Ltd, Tokyo, Japan). GE of the diet was 4.20 Mcal/kg. These diets were fed at 3% of initial body weight. The experimental period was 14 days.

Ten Holstein, lactating cows in a commercial farm were used in this study. They were evenly divided into a control group and treatment group. The cows in the control group were fed a total mixed ration (TMR). The cows in the treatment group were fed the TMR supplemented with 200 g of a commercial product (Starmate P‐50; Yuka Sangyo Co., Ltd) per head per day containing 50% of MCFA calcium soap. The cows in both group received TMR ad libitum. The experimental period was 21 days.

Ten Japanese black calves (four male and six female) in a commercial farm were used in this study. They were evenly divided into the control group and the treatment group. The calves in control group were fed a commercial starter diet. The calves in the treatment group were fed the same starter diet supplemented with 20 g of MCFA calcium soap (Starmate P‐50; Yuka Sangyo Co., Ltd) per head per day. The experiment was started just after birth and finished at 80 days after birth.

Animals were handled according to the guidelines of Mie University.

### 2.2 Sampling

Sample collection was done in a noninvasive way. Feces just voided were collected on the last day of the experiments. The feces were immediately cooled on ice and send to the laboratory and stored at −25°C. Organic acid concentration was determined using high‐performance liquid chromatography (HPLC) as described by Uddin et al. (2010). Microbial DNA was chemically extracted from feces using the QIAamp DNA Stool Mini Kit according to the manufacturer's protocol (Qiagen, Hilden, Germany). The extracted DNA was stored at −30°C until analysis.

### 2.3 Real‐time PCR assay

The cycle threshold (*C*_T_) of the following microbial populations: *Lactobacillus* spp., *Bifidobacterium* spp., *eaeA*(+) *Escherichia coli*, *Salmonella* spp. *Campylobacter jejuni*, and *Clostridium perfringens* was quantified by using real‐time PCR to determine its relative abundance. Real‐time PCR was carried out at the Life Science Research Center, Mie University (Tsu, Japan). Real‐time PCR was conducted by using the comparative ΔΔ*C*_T_ method (Schmittgen & Livak, 2008). The total bacteria were used as an endogenous control. Real‐time PCR was conducted using a StepOne Plus® Real‐time PCR System (Applied Biosystems, Foster City, CA, USA). Specific primers (5 pmol/μl) for the target group and the amount used in this study are listed in Table 1. The reaction mixture (20 μl) for real‐time PCR consisted of 1 μl of DNA template, 10 μl of Thunderbird SYBR® qPCR Master Mix (Toyobo Co., Ltd, Osaka, Japan), 0.4 μl of 50 × ROX, specific forward and reverse primers, and sterile Milli‐Q water. The PCR condition of all target groups included one cycle of polymerase activation at 95°C for 1 min, 40 cycles of denaturing at 95°C for 15 s, and annealing and extension at 60°C for 1 min. Data of the relative quantities of target groups are expressed by $2^{-\Delta \Delta C_{T}}$.

**TABLE 1:** Primers used in the present study

Target	Primer name	Nucleotide sequence (5′ → 3′)	Amount added (μl)	Reference
*Lactobacillus* spp.	R16‐1	CTTGTACACACCGCCGTA	0.2	Dubernet et al. (2002)
	LbLMAI‐rev	CTCAAACTAAACAAAGTTTC	3.6	
*Bifidobacterium* spp.	g‐Bifid‐F	CTCCTGGAAACGGGTGG	3.6	Matsuki et al. (2004)
	g‐Bifid‐R	GGTGTTCTTCCCGATATCTACA	3.6	
*eaeA*(+) *Escherichia coli*	EAE‐a	ATGCTTAGTGCTGGTTTAGGG	4.0	Fukushima et al. (2003)
	EAE‐b	GCCTTCATCATTTCGCTTTC	4.0	
*Salmonella* spp.	invA 139	GTGAAATTATCGCCACGTTCGGGCAA	4.0	Fukushima et al. (2003)
	invA 141	TCATCGCCACCGTCAAAGGAACC	4.0	
*Campylobacter jejuni*	JL238	TGGGTGCTGTTATAGGTCGT	4.0	Fukushima et al. (2003)
	JL239	GCTCATGAGAAAGTTTACTC	4.0	
*Clostridium perfringens*	GAP11	GGTTCATTAATTGAAACTGGTG	4.0	Fukushima et al. (2003)
	GAP12	AACGCCAATCATATAAATTACAGC	4.0	
Total bacteria	1114F	CGGCAACGAGCGCAACCC	0.2	Abrar et al. (2016)
	1275R	CCATTGTAGCACGTGTGTAGCC	1.2	

### 2.4 Statistics

All values are expressed as the mean and standard error in each animal. Data were subjected to a Student's *t* test. Differences of *P* < 0.05 were considered significant. Differences of *P* < 0.01 were highly significant. All statistical analyses were conducted using SPSS version 22 (SPSS Inc. Chicago, IL, USA).

## 3 RESULTS

There was no negative effect on growth performance and lactation in both treatments. Organic acid concentrations in the feces of the pigs and the lactating cow were not significantly different between the control and treatment (Table 2). In the feces of calves, propionate, butyrate, and total short‐chain fatty acids (SCFAs) were significantly lower in the treatment than in the control.

**TABLE 2:** Organic acid concentration in the feces of pigs, lactating cows, and Japanese Black calves (mmol/L)

Acid	Pigs		Lactating cows		Japanese Black calves	
	Control	Treatment	Control	Treatment	Control	Treatment
Lactate	1.5 ± 0.0	1.9 ± 0.3	1.0 ± 0.4	1.6 ± 0.5	2.1 ± 0.2	1.5 ± 0.4
Acetate	65.1 ± 5.2	70.4 ± 6.7	60.8 ± 8.3	53.9 ± 2.2	118.8 ± 4.1	99.7 ± 7.9
Propionate	22.5 ± 1.5	23.0 ± 2.0	11.8 ± 1.5	11.0 ± 0.7	37.8 ± 1.4^a^	26.2 ± 1.6^b^
Butyrate	18.4 ± 2.7	17.7 ± 1.9	8.0 ± 1.4	8.4 ± 0.6	26.4 ± 1.5^a^	16.2 ± 2.5^b^
Total short‐chain fatty acids	106.0 ± 9.0	111.1 ± 9.9	80.6 ± 11.0	73.3 ± 2.9	183.1 ± 3.5^a^	142.1 ± 11.4^b^

*Note*: Values are expressed as mean ± standard error. Different letters indicate significant differences (*P* < 0.05) between treatments.

Relative abundance of health‐promoting and pathogenic bacteria was determined (Table 3). There was no significant increase or decrease in health‐promoting bacteria in the three animals. In the pig feces, *eaeA*(+) *E. coli* was reduced to less than a third in the treatment group (*P* < 0.01). *C. jejuni* in the pig feces was also significantly less in the treatment group compared with the control (*P* < 0.01). In the lactating cow, *eaeA*(+) *E. coli* was reduced to one fifth of that in the control (*P* < 0.01). *Salmonella* spp. was halved in calf feces (*P* < 0.01).

**TABLE 3:** The effects of a medium‐chain fatty acid calcium soap on bacterial communities in the feces of pigs, lactating cows, and Japanese Black calves

Target	Pigs	Lactating cows	Calves
*Lactobacillus* spp.	1.74 ± 0.91	1.03 ± 0.22	1.10 ± 0.15
*Bifidobacterium* spp.	1.79 ± 0.56	1.73 ± 0.82	1.24 ± 0.41
*eaeA*(+) *Escherichia coli*	0.28 ± 0.04^**^	0.18^**^ ± 0.05	4.83 ± 3.25
*Salmonella* spp.	0.45 ± 0.32	0.96 ± 0.13	0.47 ± 0.08^**^
*Campylobacter jejuni*	0.62 ± 0.07^**^	3.02 ± 1.37	0.57 ± 0.20
*Clostridium perfringens*	0.89 ± 0.27	0.83 ± 0.19	3.05 ± 1.10

*Note*: Relative abundance with the control as 1.

^**^ *P* < 0.01.

## 4 DISCUSSION

SCFA concentration in the colon of weaning piglets fed diet supplemented with MCFA was not different compared with that of the piglets fed control diet (Zentek et al., 2013). Our results support this observation. There is no literature regarding to MCFA supplementation and SCFA profile in the feces of lactating cow. The present paper first showed that MCFA supplementation did not alter SCFA profile in the feces. Organic acid composition in the feces of calves was influenced by supplementation with MCFA calcium soap. The probable explanation for this result is that the supplement reduced the propionate‐ and butyrate‐producing bacteria, in the large intestine of calves. No significant effect was observed on the populations of health‐promoting bacteria such as *Lactobacillus* spp. and *Bifidobacterium* spp. in any of the animals. The use of lactobacilli in swine has been gaining attention due to their ability to improve growth performance and carcass quality and prevent gastrointestinal infection (Valeriano et al., 2017). Commercially available *Bifidobacterium* spp. probiotics maintain an optimal microbial balance of commensal bacteria against pathogenic microbes, improve growth and feed utilization, and, furthermore, enhance the gut health and intestinal immunity (Bogere et al., 2019). Microorganisms that are used in direct‐fed microbials for ruminants may be classified as lactic acid‐producing bacteria (LAB) or other microorganisms including species of *Lactobacillus* and *Bifidobacterium* (Seo et al., 2010). LAB may have beneficial effects in the intestinal tract and rumen. There is therefore no negative effect on these health‐promoting bacteria in the large intestine in the present study.

In the current study, *eaeA*(+) *E. coli* and *C. jejuni* in the feces of pigs were significantly reduced by supplementation with MCFA calcium soap. *Campylobacter* spp. are pathogens that infect to human from animal meat contaminated with the species. In the intestine of pigs, *Campylobacter coli* is a major *Campylobacter* spp. (Kelly et al., 2017). However, the present results showed that *C. jejuni* also resides in the feces of pigs. In the swine industry, postweanling diarrhea caused by enterotoxigenic *E. coli* is a globally significant enteric disease that occurs after weaning (Kim et al., 2012). *Salmonella* spp. are pathogens commonly associated with diarrhea in growing and finishing pigs (Burrough et al., 2013). Diarrhea lowers productivity of the pig. Therefore, reduction of these bacteria would result in improving porcine productivity. *E. coli* is one of the pathogenic bacteria that causes mastitis in lactating cows (Vasquez et al., 2019). Reduction in the fecal *E. coli* in lactating cows may contribute to a reduction in the incidence of mastitis.

*Salmonella* spp. are bacterial pathogens of global significance to humans and animals (Vohra et al., 2019). The pathophysiology of enteric salmonella infections is complex, involving inflammation and necrosis, increased fluid secretion, and decreased absorption and digestion (Barrington et al., 2002). In addition to enteric manifestations of disease, infected calves are frequently septicemic, which results in more severe clinical signs. Farmed animals are key reservoirs of human, nontyphoidal salmonellosis, and infections are frequently associated with ingestion or handling of contaminated meat (Vohra et al., 2019). Reduction of *Salmonella* spp. in calf feces would reduce the transfer of infection to the meat by MCFA administration.

In this experiment, a reduction in Gram‐negative pathogenic bacteria was observed. A possible explanation for these results is the antibacterial effects of MCFAs on Gram‐negative bacteria (Shibasaki & Kato, 1978). This may be due to the ability of MCFAs to diffuse into bacterial cells in an undissociated form. Inside the bacterial cell, they dissociate, lowering the intracellular pH, suppressing the cytoplasmatic enzymes and nutrient transport system, and leading to cell death (Hsiao & Siebert, 1999). However, the response varied in the animal species in this study. In this study, effects of MCFA calcium soap on Gram‐negative health‐promoting bacteria were not determined. This should be examined in a future study.

Monogastric animal and ruminants were used in the present study. Interestingly, there was no tendency within ruminants (lactating cow and calve). One of the reasons is the difference of the diet fed to the animals. Another reason for this difference is the development of the rumen.

In conclusion, supplementation of a MCFA calcium soap in the diet would be beneficial to growing pigs, lactating cow, and calves by reducing pathogenic bacteria.

## CONFLICT OF INTEREST

YY is employed by Yuka Sangyo Co., Ltd. HM, TI, MK, and TBT have no conflicts to disclose.

## References

[asj13636-bib-0001] Abrar, A. , Kondo, M. , Kitamura, T. , Ban‐Tokuda, T. , & Matsui, H. (2016). Effect of supplementation of rice bran and fumarate alone or in combination on *in vitro* rumen fermentation, methanogenesis and methanogens. Animal Science Journal, 87, 398–404. 10.1111/asj.12431 26388080

[asj13636-bib-0002] Barrington, G. M. , Gay, J. M. , & Evermann, J. F. (2002). Biosecurity for neonatal gastrointestinal diseases. The Veterinary Clinics Food Animal Practice, 18, 7–34. 10.1016/S0749-0720(02)00005-1 12064170 PMC7135474

[asj13636-bib-0003] Barton, M. D. (2000). Antibiotic use in animal feed and its impact on human health. Nutrition Research Reviews, 13, 279–299. 10.1079/095442200108729106 19087443

[asj13636-bib-0004] Bogere, P. , Choi, Y. J. , & Heo, J. (2019). Probiotics as alternatives to antibiotics in treating post‐weaning diarrhoea in pigs: Review paper. South African Journal of Animal Science, 49, 403–416. 10.4314/sajas.v49i3.1

[asj13636-bib-0005] Bomba, A. , Jonecova, Z. , Koscova, J. , Nemcová, R. , Gancarčíková, S. , Mudroňová, D. , Sciranková, L. , Buleca, V. , Lazar, G. , Pošivák, J. , Kašteľ, R. , & Mareková, M. (2006). The improvement of probiotics efficacy by synergistically acting components of natural origin: A review. Biologia, 61, 729–734. 10.2478/s11756-006-0149-y

[asj13636-bib-0006] Burrough, E. , Terhorst, S. , Sahin, O. , & Zhang, Q. (2013). Prevalence of *Campylobacter* spp. relative to other enteric pathogens in grow‐finish pigs with diarrhea. Anaerobe, 22, 111–114. 10.1016/j.anaerobe.2013.06.004 23792232

[asj13636-bib-0007] Castillo, M. , Martin‐Orue, S. M. , Taylor‐Pickard, J. A. , Pérez, J. F. , & Gasa, J. (2008). Use of mannanoligosaccharides and zinc chelate as growth promoters and diarrhea preventative in weaning pigs: Effects on microbiota and gut function. Journal of Animal Science, 86, 94–101. 10.2527/jas.2005-686 17911238

[asj13636-bib-0008] Chen, Y. J. , Kwon, O. S. , Min, B. J. , Son, S. , Cho, J. H. , Hong, J. W. , & Kim, I. H. (2005). The effects of dietary Biotite V supplementation as an alternative substance to antibiotics in growing pigs. Asian Australasian Journal of Animal Science, 18, 1642–1645. 10.5713/ajas.2005.1642

[asj13636-bib-0009] Decuypere, J. A. , & Dierick, N. A. (2003). The combined use of triacylglycerols containing medium‐chain fatty acids and exogenous lipolytic enzymes as an alternative to in‐feed antibiotics in piglets: Concept, possibilities and limitations. An overview. Nutrition Research Reviews, 16, 193–210. 10.1079/NRR200369 19087389

[asj13636-bib-0010] Desbois, A. P. , & Smith, V. J. (2010). Antibacterial free fatty acids: Activities, mechanisms of action and biotechnological potential. Applied Microbiology and Biotechnology, 85, 1629–1642. 10.1007/s00253-009-2355-3 19956944

[asj13636-bib-0011] Dubernet, S. , Desmasures, N. , & Guéguen, M. (2002). A PCR‐based method for identification of *Lactobacilli* at the genus level. FEMS Microbiology Letters, 214, 271–275. 10.1111/j.1574-6968.2002.tb11358.x 12351242

[asj13636-bib-0012] Fukushima, H. , Tsunomori, Y. , & Seki, R. (2003). Duplex real‐time SYBR green PCR assays for detection of 17 species of food‐ or waterborne pathogens in stools. Journal of Clinical Microbiology, 41, 5134–5146. 10.1128/jcm.41.11.5134-5146.2003 14605150 PMC262470

[asj13636-bib-0013] Hanczakowska, E. , Szewczyk, A. , & Okon, K. (2011). Effects of dietary caprylic and capric acids on piglet performance and mucosal epithelium structure of the ileum. Journal of Animal and Feed Science, 20, 556–565. 10.22358/jafs/66213/2011

[asj13636-bib-0014] Hsiao, C. P. , & Siebert, K. (1999). Modelling the inhibitory effects of organic acids on bacteria. International Journal of Food Microbiology, 47, 189–201. 10.1016/S0168-1605(99)00012-4 10359489

[asj13636-bib-0015] Kelly, J. , Daly, K. , Moran, A. W. , Ryan, S. , Bravo, D. , & Shirazi‐Beechey, S. P. (2017). Composition and diversity of mucosa‐associated microbiota along the entire length of the pig gastrointestinal tract; dietary influences. Environmental Microbiology, 19, 1425–1438. 10.1111/1462-2920.13619 27871148

[asj13636-bib-0016] Kim, J. C. , Hansen, C. F. , Mullan, B. P. , & Pluske, J. R. (2012). Nutrition and pathology of weaner pigs: Nutritional strategies to support barrier function in the gastrointestinal tract. Animal Feed Science and Technology, 173, 3–16. 10.1016/j.anifeedsci.2011.12.022

[asj13636-bib-0017] Kim, S. A. , & Rhee, M. S. (2013). Marked synergistic bactericidal effects and mode of action of medium‐chain fatty acids in combination with organic acids against *Escherichia coli* O157:H7. Applied and Environmental Microbiology, 79, 6552–6560. 10.1128/AEM.02164-13 23956396 PMC3811494

[asj13636-bib-0018] Liu, W. , Liu, W. L. , Liu, C. M. , Liu, J. H. , Yang, S. B. , Zheng, H. J. , Lei, H. W. , Ruan, R. , Li, T. , Tu, Z. C. , & Song, X. Y. (2011). Medium‐chain fatty acid nanoliposomes for easy energy supply. Nutrition, 27, 700–706. 10.1016/j.nut.2010.06.010 20869208

[asj13636-bib-0019] Matsuki, T. , Watanabe, K. , Fujimoto, J. , Kado, Y. , Takad, T. , Matsumoto, K. , & Tanaka, R. (2004). Quantitative PCR with 16S rRNA‐gene‐targeted species‐specific primers for analysis of human intestinal *Bifidobacteria* . Applied and Environmental Microbiology, 70, 167–173. 10.1128/aem.70.1.167-173.2004 14711639 PMC321263

[asj13636-bib-0020] Panyakaew, P. , Boon, N. , Goel, G. , Yuangklang, C. , Schonewille, J. T. , Hendriks, W. H. , & Fievez, V. (2013). Effect of supplementing coconut or krabok oil, rich in medium chain fatty acids on ruminal fermentation, protozoa and archaeal population of bulls. Animal, 7, 1950–1958. 10.1017/S1751731113001766 24237673

[asj13636-bib-0021] Piepers, S. , & De Vliegher, S. (2013). Oral supplementation of medium‐chain fatty acids during the dry period supports the neutrophil viability of peripartum dairy cows. Journal of Dairy Research, 80, 309–318. 10.1017/S0022029913000228 23570511

[asj13636-bib-0022] Schmittgen, T. D. , & Livak, K. J. (2008). Analyzing real‐time PCR data by the comparative *C* _T_ method. Nature Protocols, 3, 1101–1108. 10.1038/nprot.2008.73 18546601

[asj13636-bib-0023] Seo, J. K. , Kim, S.‐W. , Kim, M. H. , Upadhaya, S. D. , Kam, D. K. , & Ha, J. K. (2010). Direct‐fed microbials for ruminant animals. Asian‐Australasian Journal of Animal Sciences, 23, 1657–1667. 10.5713/ajas.2010.r.08

[asj13636-bib-0024] Shibasaki, I. , & Kato, N. (1978). Combined effects on antibacterial activity of fatty acids and their esters against gram‐negative bacteria. In J. J. Kabara (Ed.), The pharmacological effects of lipids (pp. 15–24). The American Oil Chemists Society.

[asj13636-bib-0025] Uddin, M. K. , Kondo, M. , Kita, J. , Matsui, H. , Karita, S. , & Goto, M. (2010). Effect of supplementation of soy sauce cake and vinegar brewer's cake with total mixed ration silage‐based diet on nutrient utilization by Holstein steers. Journal of Food Agriculture and Environment, 8, 282–287.

[asj13636-bib-0026] Valeriano, V. D. V. , Balolong, M. P. , & Kang, D.‐K. (2017). Probiotic roles of *Lactobacillus* sp. in swine: Insights from gut microbiota. Journal of Applied Microbiology, 122, 554–567. 10.1111/jam.13364 27914202

[asj13636-bib-0027] Vasquez, A. K. , Ganda, E. K. , Capel, M. B. , Eicker, S. , Virkler, P. D. , Bicalho, R. C. , & Nydam, D. V. (2019). The microbiome of *Escherichia coli* and culture‐negative non‐severe clinical mastitis: Characterization and associations with linear score and milk production. Journal of Dairy Science, 102, 578–594. 10.3168/jds.2018-15062 30447983

[asj13636-bib-0028] Vohra, P. , Vrettou, C. , Hope, J. C. , Hopkins, J. , & Stevens, M. P. (2019). Nature and consequence of interactions between *Salmonella enterica* serovar Dublin and host cells in cattle. Veterinary Research, 50, 99. 10.1186/s13567-019-0720-5 31771636 PMC6880441

[asj13636-bib-0029] Zentek, J. , Ferrara, F. , Pierper, R. , Tedin, L. , Meyer, W. , & Vahjen, W. (2013). Effects of dietary combinations of organic acids and medium chain fatty acids on the gastrointestinal microbial ecology and bacterial metabolites in the digestive tract of weaning piglets. Journal of Animal Science, 91, 3200–3210. 10.2527/jas.2012-5673 23798515

